# An enhanced workflow for variant interpretation in UniProtKB/Swiss-Prot improves consistency and reuse in ClinVar

**DOI:** 10.1093/database/baz040

**Published:** 2019-04-02

**Authors:** M L Famiglietti, A Estreicher, L Breuza, S Poux, N Redaschi, I Xenarios, A Bridge, UniProt Consortium

**Affiliations:** 1 Swiss-Prot Group, SIB Swiss Institute of Bioinformatics, CMU, Geneva 4, Switzerland; 2 University of Lausanne, Lausanne, Switzerland; 3 European Molecular Biology Laboratory, European Bioinformatics Institute (EMBL-EBI), Wellcome Genome Campus, Hinxton, Cambridge, UK; 4 Protein Information Resource, Georgetown University Medical Center, WA, USA; 5 Protein Information Resource, University of Delaware, Newark DE, USA

## Abstract

Personalized genomic medicine depends on integrated analyses that combine genetic and phenotypic data from individual patients with reference knowledge of the functional and clinical significance of sequence variants. Sources of this reference knowledge include the ClinVar repository of human genetic variants, a community resource that accepts submissions from external groups, and UniProtKB/Swiss-Prot, an expert-curated resource of protein sequences and functional annotation. UniProtKB/Swiss-Prot provides knowledge on the functional impact and clinical significance of over 30 000 human protein-coding sequence variants, curated from peer-reviewed literature reports. Here we present a pilot study that lays the groundwork for the integration of curated knowledge of protein sequence variation from UniProtKB/Swiss-Prot with ClinVar. We show that existing interpretations of variant pathogenicity in UniProtKB/Swiss-Prot and ClinVar are highly concordant, with 88% of variants that are common to the two resources having interpretations of clinical significance that agree. Re-curation of a subset of UniProtKB/Swiss-Prot variants according to American College of Medical Genetics and Genomics (ACMG) guidelines using ClinGen tools further increases this level of agreement, mainly due to the reclassification of supposedly pathogenic variants as benign, based on newly available population frequency data. We have now incorporated ACMG guidelines and ClinGen tools into the UniProt Knowledgebase (UniProtKB) curation workflow and routinely submit variant data from UniProtKB/Swiss-Prot to ClinVar. These efforts will increase the usability and utilization of UniProtKB variant data and will facilitate the continuing (re-)evaluation of clinical variant interpretations as data sets and knowledge evolve.

## Introduction

The use of patient sequence data in personalized medicine requires reference knowledge on the functional impact and role of genetic variants in human disease. The development of standardized guidelines for variant interpretation (1–4) and freely available repositories of variant data such as ClinVar (5), as well as efforts by ClinVar and ClinGen to standardize the annotation of clinical variants (6, 7) (see https://www.clinicalgenome.org/about/about-the-clingen-and-clinvar-partnership/), has been a major factor in improving the availability of reliable, high-quality genetic variant data for researchers and clinicians. Resources such as ExAC and gnomAD provide variant frequency data for large population data sets that create a framework to re-evaluate earlier pedigree studies and are another important part of ongoing efforts to standardize and improve variant interpretation (8).

UniProtKB/Swiss-Prot, the expert-curated section of the UniProt Knowledgebase (UniProtKB), contains information on over 30 000 variants linked to Mendelian diseases in 13 000 human protein sequence records (9, 10). The variant information from UniProtKB/Swiss-Prot, curated from decades of peer-reviewed literature, represents an important resource for understanding the impact of protein sequence variants. The additional context provided by curated information on protein function, subcellular location, interactions and sequence features, such as protein domains, active sites and post-translational modifications, can also help to inform the analysis of the role of sequence variants in disease.

Here we present a pilot study that aims to facilitate the reuse of curated variant data from UniProtKB/Swiss-Prot by ClinVar, thereby laying the groundwork for further integration of these two resources. We find that existing variant annotations that are common to the two resources are highly consistent and that consistency is further improved by re-curation of UniProtKB/Swiss-Prot variants using ClinGen tools for variant interpretation and guidelines provided by the American College of Medical Genetics and Genomics and the Association for Molecular Pathology (ACMG-AMP, abbreviated here as ACMG) (2). Our work confirms the feasibility and utility of incorporating ClinGen guidelines and tools into the UniProtKB curation workflow, and we now routinely submit newly curated standardized interpretations of variant data from UniProtKB/Swiss-Prot to ClinVar. This development will facilitate the reuse and continuous (re-)evaluation of variant annotations from UniProtKB/Swiss-Prot and other sources as new knowledge becomes available.

## Materials and methods

### Identifying variants shared by UniProtKB/Swiss-Prot and ClinVar and generation of working sets for variant reinterpretation

ClinVar accepts variant interpretations from clinical testing laboratories, research laboratories, locus-specific databases, expert panels and other groups. Submissions that deal with the same variant and variant–phenotype relations are grouped in a single ClinVar record, which is assigned a defined review status score. The scores and their meanings are as follows: 0 star, interpretations by a single submitter without any assertion criteria; 1 star, interpretations by a single submitter with assertion criteria provided; 2 stars, concordant interpretations by multiple submitters with assertion criteria provided; 3 stars, interpretations by expert panels; and 4 stars, interpretations by clinical practice guideline-providing groups. In this work, we compared UniProtKB/Swiss-Prot variant interpretations with those from other submitters found in ClinVar records having 2-star status.

Using UniProtKB and ClinVar releases of December 2017, we used common dbSNP identifiers and Human Genome Variation Society (HGVS) names to identify 4286 missense variants in UniProtKB/Swiss-Prot that were also reported in the ClinVar records with 2-star status (Supplementary Table S1). As ACMG guidelines are only applicable to variants associated with Mendelian diseases, we excluded those variants found in multifactorial disorders. We selected subsets of variants for re-curation randomly from this set of 4 286 common variants.

### Defining agreement in interpretations of clinical significance

ClinVar employs the 5-tier classification developed by ACMG for variants associated with Mendelian diseases; a specific variant may be classified as pathogenic, likely pathogenic, benign, likely benign or of uncertain significance with respect to a particular disease (2). UniProtKB classifies variants associated with Mendelian diseases as either disease (causing), polymorphism or unclassified with respect to a particular disease (10). We define equivalence between the two classification schemes as follows: UniProtKB/Swiss-Prot interpretation `disease’ corresponds to ClinVar `pathogenic’ and `likely pathogenic’, `polymorphism’ corresponds to `benign’ and `likely benign’ and `unclassified’ corresponds to `uncertain significance’ (see http://www.uniprot.org/docs/humsavar).

### Calculating concordance between interpretations of clinical significance

For a given category of variants, we define the percentage of concordance between UniProtKB/Swiss-Prot and ClinVar as the number of interpretations common to both resources, divided by the number of total interpretations in either of the two resources, multiplied by 100. For instance, concordance for variants interpreted as polymorphisms in UniProtKB/Swiss-Prot (likely benign or benign in ClinVar) is 100 × (2328 / 2328 + 46 + 128 + 111 + 81) or 86% (see Table 1).

**Table 1:** Global comparison of the interpretations of 4286 variants common to UniProtKB/Swiss-Prot and ClinVar

Original classification in UniProtKB/Swiss-Prot	Classification in ClinVar			Total
	P/LP	B/LB	VUS	
Disease	1328	111	94	1533
Polymorphism	46	2328	128	2502
Unclassified	55	81	115	251
Total	1429	2520	337	4286

P/LP, pathogenic/likely pathogenic; B/LB, benign/likely benign; VUS, variant of uncertain significance. Diagonals represent concordant interpretations, off-diagonals discordant interpretations.

### Tools and variant interpretation procedure

We used the ClinGen pathogenicity calculator (11) to re-curate UniProtKB/Swiss-Prot variants according to ACMG guidelines. This tool allows users to enter evidence for a specific variant–disease association with links to supporting data. The tool assigns a default weighting to each evidence (which can be modified if required) and combines evidences to generate a report with the appropriate pathogenicity assessment. Information on diseases, variants and functional data was gathered from peer-reviewed publications and other resources, including OMIM (12), Genetics Home Reference (13), Orphanet (14), the Exome Aggregation Consortium (ExAC release 0.3) and the genome Aggregation Database (gnomAD release 2.0) (8). Allele frequency cutoffs (to evaluate variant frequency) were obtained from ExAC. The maximum credible population allele frequency was calculated using online tools (15) when disease prevalence information was available. Computational evidence was taken into account if the predictions from PolyPhen (16), SIFT (17) and MutationTaster (18) were concordant.

## Results

### Global comparison of variant interpretation

We first analyzed 4286 interpretations of variant pathogenicity common to UniProtKB/Swiss-Prot and ClinVar 2-star records. We reasoned that comparison of variants from UniProtKB/Swiss-Prot with the set of ClinVar 2-star records—where multiple submitters agree on the interpretation of clinical significance, but which lack validation by expert panels—is likely to provide an easily interpretable upper-bound estimate of the level of disagreement between UniProtKB/Swiss-Prot and ClinVar.

A total of 3771 interpretations of variant pathogenicity assessments (88%) were found to be in agreement between UniProtKB/Swiss-Prot and ClinVar 2-star records (Table 1; a complete listing of all variant interpretations is provided in Supplementary Table S1). Concordance was 86% for variants interpreted as polymorphisms in UniProtKB/Swiss-Prot (likely benign or benign in ClinVar), 81% for disease variants from UniProtKB/Swiss-Prot (likely pathogenic or pathogenic in ClinVar) and only 24% for variants of uncertain significance (see Table 1). Variants classified as being of uncertain significance in either resource are over-represented in the set of variants whose interpretations disagree, possibly because the individual lines of evidence about these variants are inconclusive in isolation and so particularly susceptible to varying interpretation. We provide an example to illustrate this below.

### The effect of re-curation on variant interpretation between ClinVar and UniProtKB/Swiss-Prot

To investigate the effect of re-curation on the level of agreement of variant interpretations in UniProtKB/Swiss-Prot and ClinVar, we randomly selected two sets of variants where the interpretations of variant pathogenicity in UniProtKB/Swiss-Prot and ClinVar either agreed (100 variants) or disagreed (100 variants). Each set was re-curated using the ACMG guidelines and the ClinGen pathogenicity calculator.

In the set of 100 discordant variants, we solved 78 conflicts, modified 7 interpretations without reaching agreement with ClinVar and retained 15 of the existing interpretations (Table 2). The most common change observed upon re-curation of discordant variants was the reclassification of pathogenic variants as benign variants or variants of uncertain significance, due to the great majority of cases to the use of population data.

**Table 2:** Re-curation of 100 UniProtKB variants with discordant interpretations compared to ClinVar

Original classification in UniProtKB/Swiss-Prot	Classification following re-curation			Total
	P/LP	B/LB	VUS	
Disease	1	31	20	52
Polymorphism	6	4	8	18
Unclassified	4	16	10	30
Total	11	51	38	100

P/LP, pathogenic/likely pathogenic; B/LB, benign/likely benign; VUS, variant of uncertain significance.

In the set of 100 concordant variants, we confirmed 96 interpretations but modified the interpretation of 4 variants, introducing 4 new conflicts between the two resources (Table 3). The most common change observed upon re-curation of concordant variants was the reclassification of pathogenic variants as variants of uncertain significance.

**Table 3:** Re-curation of 100 UniProtKB variants with concordant interpretations compared to ClinVar

Original classification in UniProtKB/Swiss-Prot	Classification following re-curation			Total
	P / LP	B / LB	VUS	
Disease	54	0	4	58
Polymorphism	0	38	0	38
Unclassified	0	0	4	4
Total	54	38	8	100

P/LP, pathogenic/likely pathogenic; B/LB, benign/likely benign; VUS, variant of uncertain significance.

The reduction in the number of variants interpreted as `(likely) pathogenic’ and the increase of the number of `uncertain significance’ and `(likely) benign’ in both sets of 100 randomly selected variants are in line with the findings of others (8, 19).

We describe below two representative examples from this re-curation work. The first example illustrates how the use of newly available population frequency data results in the reclassification of a supposedly pathogenic variant as `likely benign’. The reclassification of pathogenic variants as benign or likely benign is the most common change seen upon re-curation in our study and is the main cause of improved agreement in variant interpretations. The second example illustrates how functional data can inform consideration of population frequency data. In this particular case, this leads to reclassification of a pathogenic variant as being of `uncertain significance’ and creates a conflict between UniProtKB/Swiss-Prot and ClinVar—which is relatively common for variants of `uncertain significance’. This highlights how only coordinated re-curation of interpretations of variant pathogenicity by multiple submitters will allow the community to achieve agreement on these difficult cases.

### Example 1. Population frequency data support reclassification of a pathogenic variant in *GLI3* as likely benign, in agreement with ClinVar.

Transcriptional activator *GLI3* (UniProtKB accession P10071) mediates Hedgehog (Hh) signaling in embryogenesis and limb development (20). *GLI3* pathogenic variants cause non-syndromic and syndromic polydactyly, including the rare autosomal dominant Greig cephalopolysyndactyly syndrome (GCPS, OMIM accession 175700) (21, 22). GCPS has an estimated prevalence of around 1 in 1 000 000 in the general population (23).

*GLI3* variant p.Ile808Met (NM_000168.5:c.2424A>G; ClinVar Variation ID 235210) was originally annotated in UniProtKB/Swiss-Prot as a pathogenic variant causing GCPS, based on small-scale pedigree studies (24) and functional assays showing a deleterious effect of this variant on nuclear localization and transcriptional activity (25). Multiple submitters to ClinVar had annotated this variant as `Benign’ or `Likely benign’ for GCPS based on the results of clinical testing.

Re-curation of this variant in UniProtKB/Swiss-Prot led to the resolution of this discrepancy; the variant was reclassified as `Likely benign’ as the observed variant allele frequencies (close to 0.2% in ExAC and gnomAD) are not consistent with a pathogenic role in GCPS (which has a much lower estimated prevalence in the population). The ACMG guidelines recommend to `consider how closely a functional assay reflects the biological environment’ when using functional data for variant interpretation. We therefore chose in this case to disregard the previous functional data on this variant due to the lack of a clear mechanistic link between *GLI3* functional impairment observed *in vitro* and the disease. The precise ACMG criteria that are relevant in this case include the following: `Allele frequency is greater than expected for disorder’ (abbreviated to `BS1’) and `Observed in a healthy adult individual for a recessive (homozygous), dominant (heterozygous), or X-linked (hemizygous) disorder, with full penetrance expected at an early age’ (`BS2’). Both could be considered as `strong’ evidence that this variant is benign, but we considered `BS2’ only as `supporting’ evidence as GCPS penetrance is not 100% (23).

### Example 2. Conflicting functional and population data support reclassification of a pathogenic variant in *ATP7B* as being of uncertain significance, creating a disagreement with ClinVar.

*ATP7B* (UniProtKB accession P35670) is a transmembrane ion transporter involved in cellular copper ion homeostasis that is predominantly expressed in the liver (26). *ATP7B* dysfunction causes Wilson disease (WD, OMIM accession 277900), an autosomal recessive disorder characterized by decreased biliary excretion of copper, impaired copper incorporation into ceruloplasmin (the major copper carrying protein in the blood) and copper accumulation in the liver, brain, kidney and eye. Disease penetrance is incomplete, and clinical presentations are highly variable, mainly consisting of hepatic and neurological symptoms. WD prevalence is estimated to be 1/30 000 with a carrier frequency of 1.1% (1/90) according to Genetics Home Reference and the medical literature (27), although some authors report a prevalence of 1/10 000 or higher as many patients may remain undiagnosed (28, 29).

*ATP7B* variant p.Met645Arg (NM_000053.3:c.1934T>G; ClinVar Variation ID 3862) was originally interpreted as pathogenic in both UniProtKB/Swiss-Prot and ClinVar. The interpretation in UniProtKB/Swiss-Prot was based on published claims of pathogenicity and the repeated identification of this variant in patients with WD, often in a compound heterozygous state with a pathogenic variant (27, 30–34). Other ClinVar submitters have interpreted the clinical significance of this variant as pathogenic, based on the results of clinical testing.

Re-curation of this variant in UniProtKB/Swiss-Prot led us to reclassify this variant as of `uncertain significance’ for WD due to conflicting population and functional data, thereby creating a new discrepancy between UniProtKB/Swiss-Prot and other ClinVar submitters. Population data argue for a role for this variant in WD: the overall frequency of p.Met645Arg is 0.05% in ExAC, a value well below the known carrier frequency, and no homozygote is reported in ExAC, nor in gnomAD. However, copper transport activity of p.Met645Arg is comparable to that of the wild-type protein (35, 36), and the variant is also found in a compound heterozygous state in a patient with hypoceruloplasminemia, devoid of any clinical manifestations of WD and with normal copper levels in Kupffer cells and hepatocytes (37). The ClinGen pathogenicity calculator provides three distinct and conflicting ACMG evidence types for these findings. `Absent from controls (or at extremely low frequency if recessive) in Exome Sequencing Project, 1000 Genomes Project, or Exome Aggregation Consortium’ (PM2), `For recessive disorders, detected in trans with a pathogenic variant’ (PM3) and `Well-established *in vitro* or *in vivo* functional studies show no damaging effect on protein function or splicing’ (BS3).

## Discussion

Here we show that interpretations of the pathogenic significance of human sequence variants are highly concordant between UniProtKB/Swiss-Prot and ClinVar, with 88% of interpretations agreeing. Our re-curation work using ACMG guidelines and ClinGen tools suggests that agreement could be expected to improve even further, probably to around 94% for all UniProtKB/Swiss-Prot variants found in ClinVar. Re-curation leads to a decrease in the proportion of UniProtKB/Swiss-Prot classified as `pathogenic’, which is due mainly to the use of newly available population frequency data from resources such as ExAC and gnomAD. These resources provide much deeper coverage of human sequence variation than the small-scale pedigree analyses on which many of the original interpretations were based. Other studies that analyzed ClinVar submissions from multiple independent clinical laboratories have reported similar findings (7, 38).

The ACMG guidelines and the ClinGen pathogenicity calculator provide a framework for consistent curation that serves to focus curator judgment, but curator judgment remains essential to choose and appropriately weight ACMG-based evidences, as illustrated by the two cases presented here. *GLI3* is a transcriptional activator mediating Hh signaling in embryogenesis and limb development. Experimental assays performed *in vitro* suggest that *GLI3* variant p.Ile808Met reduces *GLI3* transcriptional activity to some degree, but there is no information on the effect on `Hh’ signaling. Available data do not meet the ACMG recommendations for the use of functional evidence. Published guidelines also recommend the consideration of functional information as an auxiliary support to genetic data (1, 2), and very strong population data indicates that this variant is benign with respect to GCPS. We therefore chose to disregard the available *in vitro* assay data for the purposes of assessing this variant, as this would have introduced unfounded uncertainty about the role of this variant in this disease. This contrasts with how functional data was used in our reinterpretation of the role of *ATP7B* variant p.Met645Arg in WD. Here, convincing experimental data using a variety of assays find no effect of p.Met645Arg on *ATP7B* copper transport activity *in vitro* or *in vivo*. These findings affect consideration of genetic evidence, where allele frequency is compatible with a role in disease. One explanation for this apparent discrepancy in genetic and functional data could be that *ATP7B* variant p.Met645Arg is in linkage disequilibrium with an as yet unidentified pathogenic variant defining a complex allele. In this scenario, variant p.Met645Arg would be recurrently detected in WD patients but would exhibit no functional effect when tested in isolation.

We could not solve all conflicts between the subset of UniProtKB/Swiss-Prot variants analyzed here and variants in ClinVar, but we do not expect to be able to achieve complete agreement without working together with other ClinVar submitters. Yang *et al.* (38) found that most submitters agreed on around 90% of variant classifications and that in general one or more outliers (mostly research and curation groups) provided a different interpretation from the rest. Variants of uncertain significance are highly represented among the set of variants with interpretations that disagree in UniProtKB/Swiss-Prot and ClinVar. One source of these discrepancies could be in the way groups use functional data, as illustrated by the case of *ATP7B* variant p.Met645Arg, where functional and population data seem to say different things about the possible role of this variant in disease. Groups that do not consider functional data from the literature will classify this variant differently from those who do, and for whom there is more doubt about its role, while the weight given to functional evidence also strongly influences variant interpretation. Others have also found that the use of functional data is a major source of persistent differences in variant interpretation between groups (7).

Another source of possible inconsistency is the use by clinical laboratories of private data and guidelines (particularly for specific diseases) that differ from the general guidelines of ACMG [e.g. (39)]. Even when interpretations are unanimous, they may not necessarily be correct, and systematic review by experts using multiple evidence types (functional and genetic) is likely to be the best guarantee of accurate assessments of pathogenicity. In this setting, the role of the biocurator may be to draw expert attention to conflicting evidence and variant interpretations that deserve re-examination by clinicians, as in the case of 21 genes implicated in Brugada syndrome, of which 20 were reclassified as disputed with regard to any assertions of a pathogenic role by a clinical domain expert panel (40).

Efforts to standardize variant interpretations using shared guidelines and tools promote interoperability and data sharing between resources. We have now incorporated ACMG guidelines and the ClinGen pathogenicity calculator in our daily curation workflow for UniProtKB/Swiss-Prot and routinely submit our interpretations of variant pathogenicity to ClinVar. We will continue to re-curate existing variant interpretations in UniProtKB/Swiss-Prot, particularly variants whose interpretations in UniProtKB/Swiss-Prot differ from those found in ClinVar, as well as curating newly discovered variants. We expect that these efforts will facilitate the reuse of our data and increase the already high degree of concordance between UniProtKB/Swiss-Prot and ClinVar.

With the increasing amount of variation data generated by massive widespread genome sequencing, the scientific community is just at the beginning of a huge effort to understand the role of genetic variants in health and disease. While discerning only the tip of the iceberg, one may hear the philosopher saying `I know that I know nothing’.

**Links**

UniProtKB: https://www.uniprot.org

Humsavar: https://www.uniprot.org/docs/humsavar

Allele frequency calculator: http://cardiodb.org/allelefrequencyapp

ClinGen: https://www.clinicalgenome.org

ClinGen pathogenicity calculator: https://calculator.clinicalgenome.org/site/cg-calculator

ClinVar: https://www.ncbi.nlm.nih.gov/clinvar

ExAC: http://exac.broadinstitute.org

Genetics Home Reference: https://ghr.nlm.nih.gov

gnomAD: http://gnomad.broadinstitute.org

OMIM: https://www.omim.org

Orphanet: https://www.orpha.net/consor/cgi-bin/index.php

Submissions to ClinVar made in the course of this work can be found at https://www.ncbi.nlm.nih.gov/clinvar?term=SIB[Submitter].

## Supplementary Material

Famiglietti_SupplementaryTableS1_REV_baz040

## Notes

**Database URL**: www.uniprot.org/
